# Restoring visual capacity after stroke using an intense office‐based vision therapy program: Three case reports

**DOI:** 10.1002/ccr3.2064

**Published:** 2019-02-22

**Authors:** Ingrid Axelsson, Anders Holmblad, Jan Johansson

**Affiliations:** ^1^ Stockholm Low Vision Center Stockholm Sweden; ^2^ Department of Clinical Neuroscience, Eye and Vision Karolinska Institutet Stockholm Sweden

**Keywords:** eye‐tracking, stroke, vision therapy, visual field

## Abstract

Visual function problems is a common, yet easily overlooked group of issues after stroke that may hamper the capacity to perform rehabilitation activities and resume daily tasks. We show the potential of an intense vision therapy program for restoring objectively measurable visual functions and documented improvements in activity‐based performance.

## 1 INTRODUCTION

We describe the functional and activity‐based outcomes from an intense vision therapy program targeting visual function issues, including issues related to peripheral visual field loss, following stroke. Three case reports are presented and the outcome measures include self‐assessment of performance, clinical measures of visual function, and eye‐tracking.

Functional deficits after stroke may cause a constellation of symptoms and issues that either directly or indirectly limit the capacity to resume daily tasks. Visual function deficits are frequently part of the issues including reduced visual acuity, peripheral visual field loss, eye movement‐ and visual‐perceptual deficits.^1^ These issues obviously have direct effects on visually demanding tasks such as reading, computer work, orientation, and interacting with other people.^2^ Visual function may also take an even more important role after the injury since it is necessary to find ways of compensating for loss of visual field or decreased sensory‐motor functions needed for postural stability and mobility. Ensuring that visual function issues are detected and intervened is therefore of great importance for creating optimum conditions in the rehabilitation process.

This case report describes the outcome of vision therapy for three patients who had been referred mainly due to peripheral visual field related issues. The extent of visual field loss had been established with automated visual field testing at the ophthalmological assessment previous to clinic admittance. At the initial assessment comprehensive examinations were performed by a vision therapist (VT) and a licensed optometrist (LO) to establish status of visual acuity, visual fields, binocular functions, gaze stability (fixation), and gaze shifts (saccades, smooth pursuit). In addition, the activity‐based performance was estimated using the Canadian Occupational Performance Measure (COPM).^3^ Reading‐related performance was assessed using the Pepper Visual Skills Test for Reading Test (VSRT)^4^ and International Reading Speed Test (IReST).^5^

The vision therapy program (VTP) included 10 one‐hour sessions evenly distributed over a four‐week period. The deficits were targeted with customized, specific vision therapy activities in one‐to‐one sessions assisted by the vision therapist and the optometrist. Vergence‐related deficits were targeted with free space training (Brock's string, Hart chart, Eccentric circles, Three cats) and computerized training using anaglyphs (Vision‐builder.no). Fixation and saccades were targeted with systematic exercises requiring the patient to make precise eye movements and quickly achieve a steady fixation (Hart chart, Saccadic strips). Visual search training included timed tasks with the goal of identifying objects with a certain criteria (Dice, Alphabet cards, color‐coded cards). Common to the eye movement training activities was an emphasis on keeping the head still and to use eye movements. Compliance to this was ensured through monitoring and feedback by the vision therapist. Each training activity was performed for 5‐10 minutes with a short pause before continuing with the next. The sequence of training activities was adapted to alter continuously between activities performed seated and standing. In our experience this continuous variation promotes the patient's perseverance during the session.

Eye movements during fixation and saccade test paradigms (Table A1) were recorded before and after VTP using an eye‐tracker (SMI‐Red 250, www.smivision.com). Recordings from age (±2 years) and gender matched controls were used for reference. Consent was obtained from all three patients and the controls.

## 2 CASE PRESENTATIONS

### 2.1 Case #1

A 29‐year‐old female patient was referred for visual function assessment 3 months after a left sided occipital infarct. The main symptoms were feeling insecure while ambulating outdoors, fatigued by visually stimulating environments, and slow reading with poor perseverance. The assessment showed right‐sided homonymous hemianopsia without macula sparing. Visual acuity was decimal 1.3 (Sph. plano, cyl. −0.75 D @ 180 degrees) for right eye (RE) and left (LE). Near point of convergence (NPC) and accommodation (NPA) was in accordance with expected normal values^6^ but positive fusion vergence was reduced and vergence facility could not be performed (Table 1).

**Table 1:** Clinical and activity‐based measures for patient #1. Only measures with subnormal values before VTP are presented

	Before VTP		After VTP		
*Clinical measures*					*Comment*
Positive fusional vergence	6 Base Out		30 Base Out		Normalized
Vergence facility (3 base in /12 base out)	0 cycles		15 cycles		Normalized
*Eye movement recordings*					*Control #1*
Fixation, conf. ellipse area	4.23		1.06		0.79
Pro‐saccade latency, rightward/leftward ms	748/241.8 ± 110.5		214.1 ± 56.2/211.3 ± 55.3		212.1 ± 39.7/206.5 ± 35.0
*Activity‐based measures*					*Comment*
VSRT test	59 wpm		75 wpm		+27.1%
IReST reading speed	114 wpm		143 wpm		+25.4%
*COPM*	*Performance*	*Satisfaction*	*Performance*	*Satisfaction*	*Comment*
Ambulation (importance 10)	5	1	8†	7†	†Clinically significant improvement
Reading (importance 8)	3	1	4	5†	†Clinically significant improvement

ms: milliseconds; wpm, words per minute.

**Figure 1:**
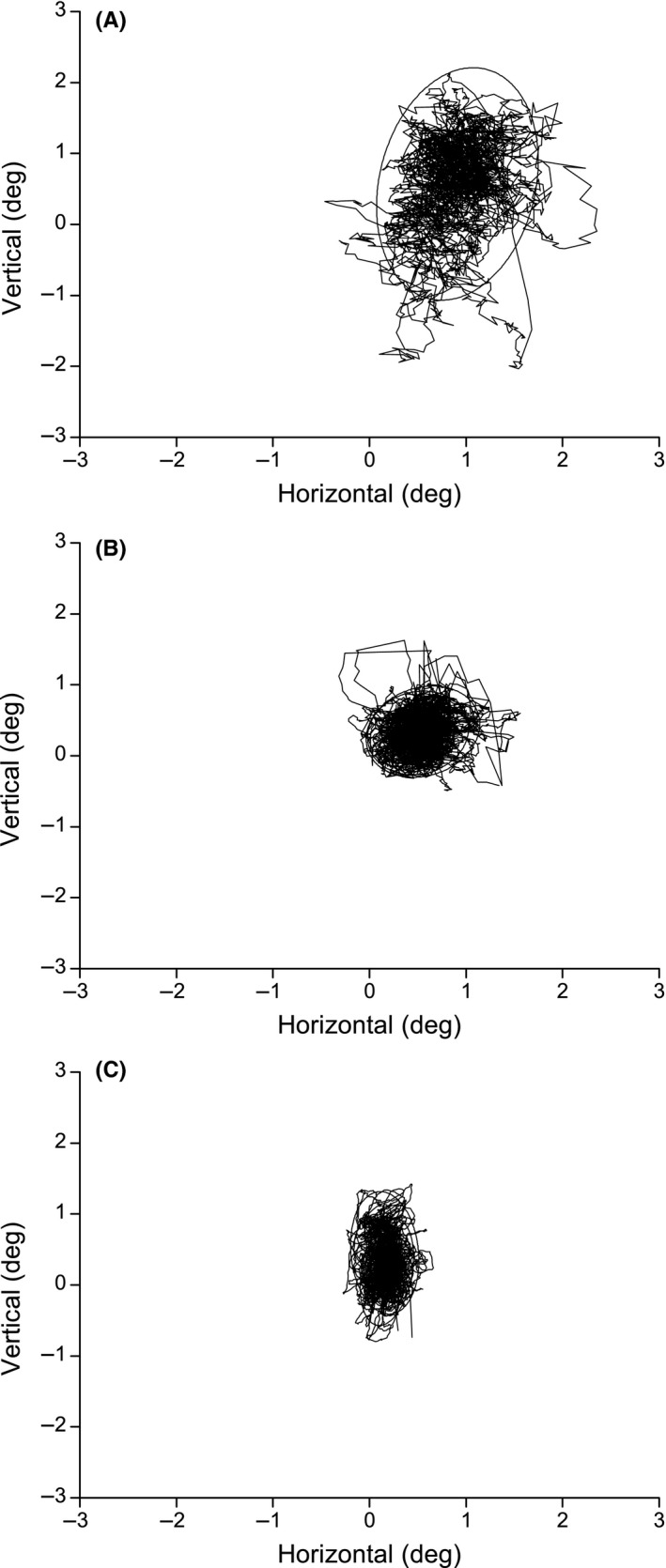
Fixation pattern in patient #1 before (A) and after (B) vision therapy program (VTP) compared to control #1 (C). The plots show horizontal (*x*‐axis) and vertical (*y*‐axis) eye position in degrees where zero depicts the position of the stimulus dot. The signal of RE is excluded in (A) due to poor signal quality. The area of the confidence ellipse decreased after VTP (Table 1) indicating a less variable fixation behavior

**Figure 2:**
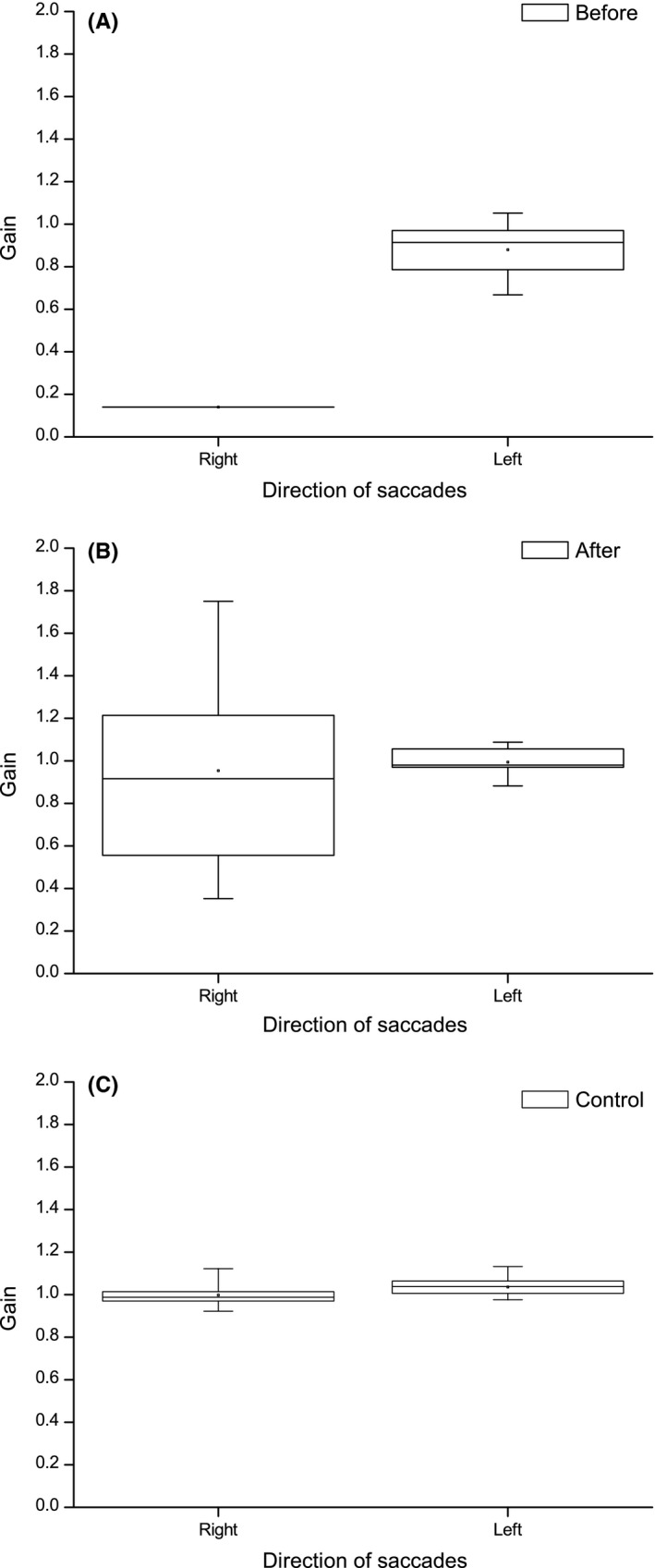
Pro‐saccade gain performance in patient #1 before (A) and after (B) vision therapy program (VTP) compared to control #1 (C). Note that only one rightward saccade was performed before VTP while after VTP, all but two (8.3%) were performed successfully. The box charts indicates 25, 50, and 75 percentiles and whiskers indicate min and max values. The miniature squares indicates mean values

The VTP included convergence, fusional vergence, and saccadic exercises. After completing the program, the patient described an improved visual perseverance despite remaining mental fatigue. Most of the daily news could now be read as opposed to just single news items before. An improved automaticity in eye movements toward the right, with less need for head movement, was also described. The clinical, eye movement, and activity‐based measures showed improvements as described in Table 1. Eye movement recordings showed an improvement in fixational stability (Figure 1) and pro‐saccade gain performance (Figure 2).

### 2.2 Case #2

This case regards another female patient of age 29 years who had suffered a right‐sided occipital infarction 6 months previous to clinic admittance. The main symptom was a marked discomfort while ambulating in public environments due to a worry of tripping over objects or bumping into people. The scanning of the environment was mainly achieved through head movements. Reading performance was not perceived as a major concern. The visual function assessment showed a normal corrected visual acuity of decimal 1.3 (Sph −0.50 D) for RE and LE, normal NPC (5 cm), better than normal accommodative amplitude (12 D). However, positive fusional vergence was low (six base out), vergence facility was zero, stereo acuity was TNO 120, and smooth pursuit eye movements toward the left, and particularly up‐left, was hampered with several re‐fixations. The COPM assessment identified problems with ambulation as a major concern (importance 10, performance 6, satisfaction 2) due to problems with attention and visual scanning to the left.

The VTP included convergence, fusional vergence, and saccadic eye movement exercises with individual coaching and feedback by the VT. The post‐VTP evaluation showed clinically significant improvements in COPM with an increase by two points in performance and by six points in satisfaction. The patient described an improved awareness, efficiency, and perseverance in the scanning behavior. Eye motility testing showed an improved smooth pursuit performance with minimal re‐fixations. Fusional vergence and vergence facility data were not available from the follow‐up examination; however, a markedly improved stereo acuity (TNO 30) suggested an improved sensitivity to disparity cues which is an important component for adequate vergence responses.

**Figure 3:**
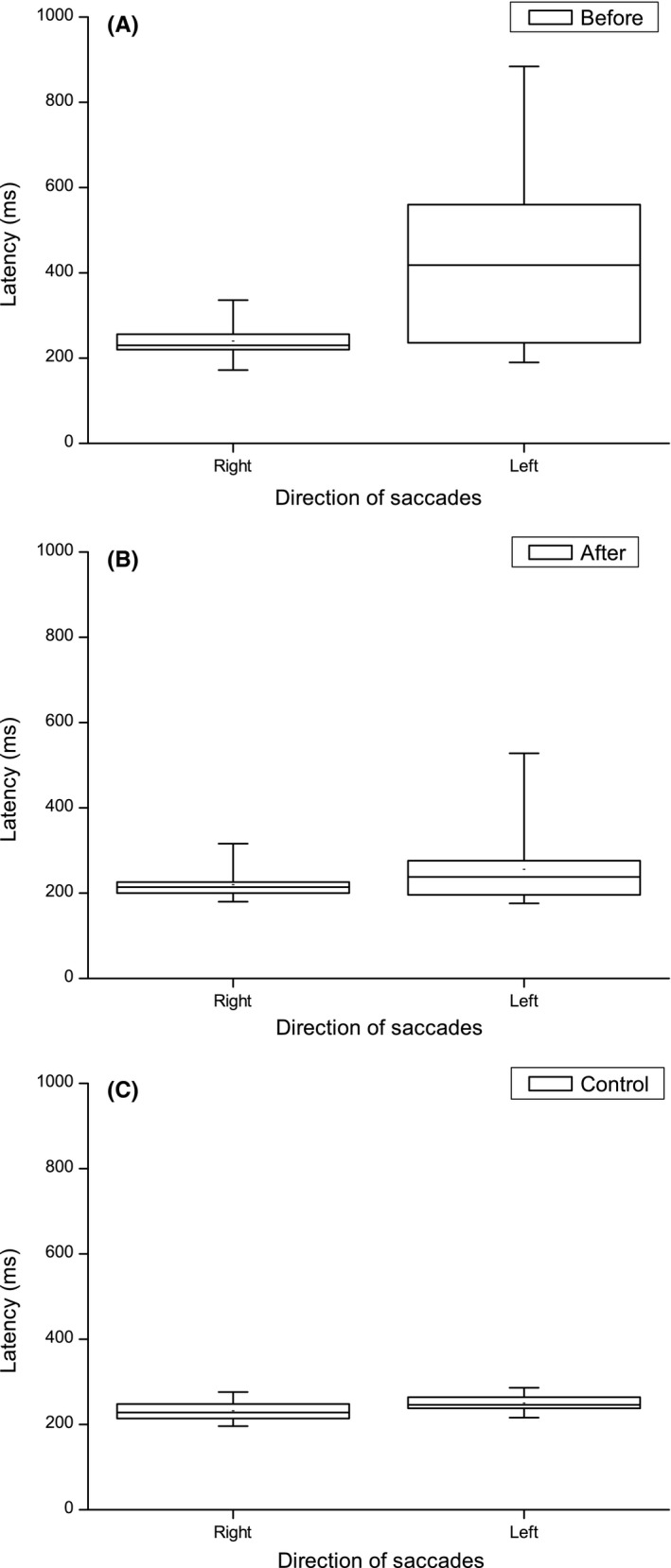
Pro‐saccade latency performance in patient #2 before (A) and after (B) vision therapy program (VTP) compared to control #2 (C). Before VTP, the mean latency for leftward saccades was longer and more variable compared to rightward. After VTP, this difference evened out with no statistically significant difference remaining

Eye movement analysis showed that before VTP, the leftward pro‐saccade latency was significantly prolonged compared to rightward (Student's *t* test, *P* = 0.025). After VTP, the latency for leftward saccades had improved to the point that no statistically significant difference remained (Figure 3).

The gain of pro‐saccades before VTP was for rightward saccades 0.8 ± 0.3 and for leftward 0.7 ± 0.2 (nonsignificant difference). After VTP, rightward was 1.0 ± 0.2 and leftward 0.8 ± 0.2 which was a statistically significant difference (Wilcoxon signed rank test, *P* = 0.005).

**Figure 4:**
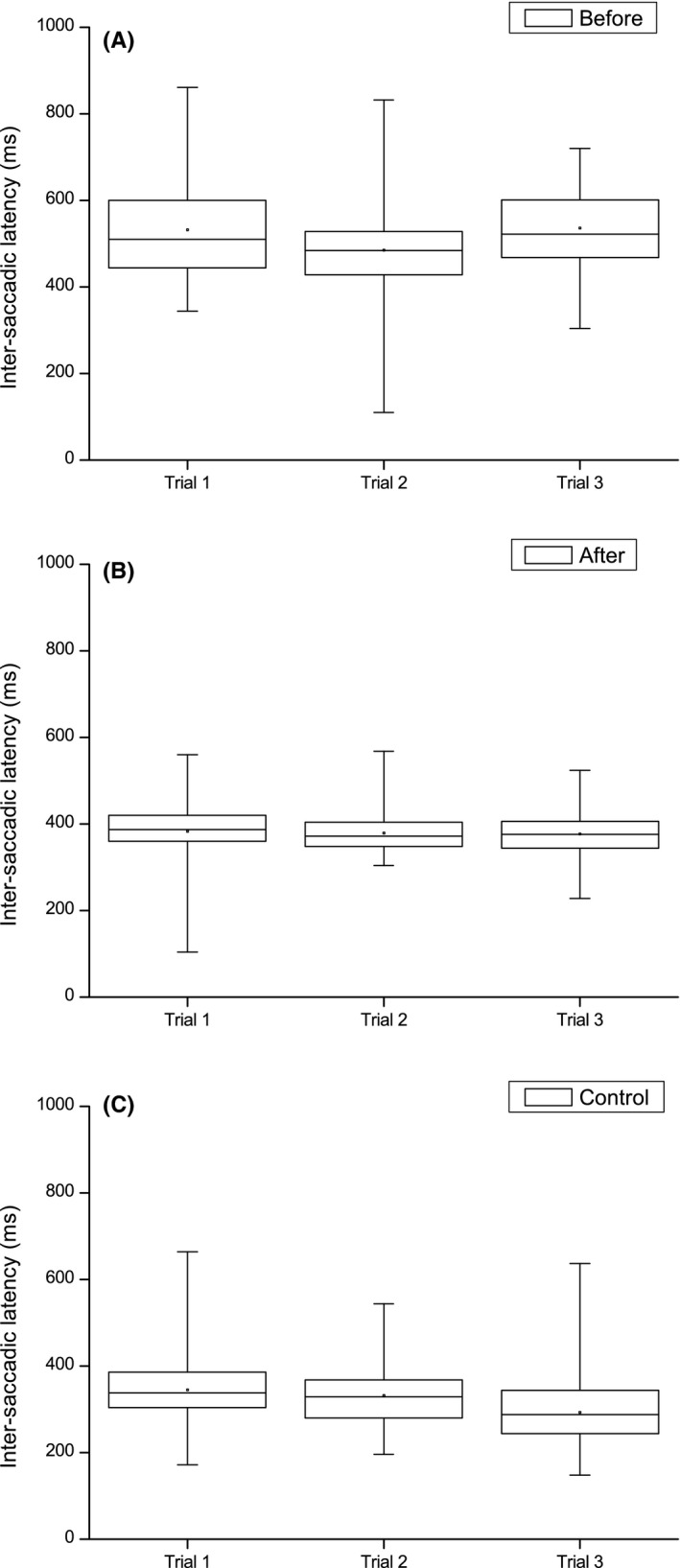
The mean intersaccadic latency for patient #2 before (A) and after vision therapy program (VTP) (B) with control #2 (C) as reference. The self‐paced saccade test was performed three times in series at each occasion (Trial 1‐3)

The self‐paced saccades test was performed three times in series before and after VTP (Figure 4). The mean intersaccadic latency (ISL), the time between one saccade was finished and the next initiated, was significantly shorter after VTP (380.0 ± 54.4 ms) compared to before (517.7 ± 115.7 ms) (Welch's *t* test, *P* = 0.000). The ISL was now closer to the performance of the control (323.4 ± 75.0) but the difference was still statistically significant (Welch's *t* test, *P* = 0.000).

### 2.3 Case #3

This case concerns a female patient, age 62, where VTP was initiated 16 months postinjury (bilateral occipital infarction). The patient described considerable difficulties including balance issues, being surprised by sudden appearance of people coming from the right hand side, bumping into people and objects, and quickly fatigued when trying to read. The COPM assessment identified orientation/ambulation (importance 10, performance 4, satisfaction 3) and reading (importance 9, performance 5, satisfaction 5) as the main concerns. The visual examination showed right‐sided homonymous hemianopia with macula sparing, scotomas in upper left quadrants, and a receded near point of convergence (17 cm). Corrected visual acuity was decimal 0.9 for RE (Sph. +0.50 Cyl. −1.00 @ 95 deg) and LE (Sph. −0.75 Cyl. −1.00 @ 75 deg). The result of Bell's test for visual scanning was 24/35 indicating that 31% of objects were left out. The VSRT test result was 63 wpm and IReST 122 wpm.

The VTP focused on fixation, saccade, visual search, and reading exercises. After VTP, the COPM scores for orientation/ambulation performance improved by two points (clinically significant), and for reading, it improved by two points both in performance and satisfaction. The Bell's test result was now 34/35 (97%), VSRT 80 wpm (+27%), and IReST 143 wpm (+17%). The patient described an improved insight in the visual function, an improved efficiency and perseverance in visual scanning, and less bumping into people and objects. She also perceived an improved confidence while ambulating due to better steadiness and awareness of surrounding environment.

**Figure 5:**
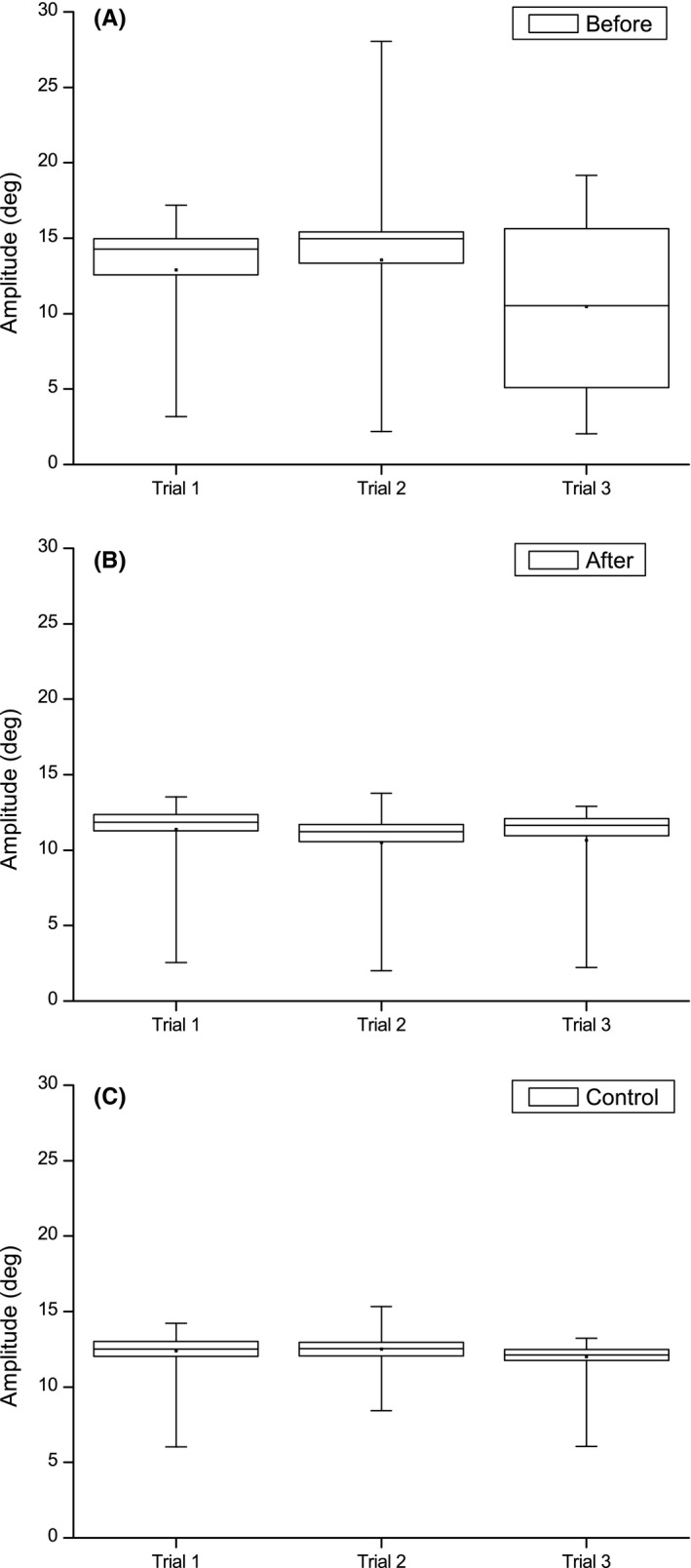
Self‐paced saccade amplitude performance for patient #3 before vision therapy program (VTP) (A) and after (B) compared to control #3 (C). The ideal amplitude is 12 degrees since the stimuli were separated horizontally ±6 degrees. The self‐paced saccade test was performed three times in series at each occasion (Trial 1‐3)

The mean ISL was somewhat lower after VTP (372.2 ± 99.8 ms) compared to before (440.2 ± 122.3 ms) and closer to the performance of control #3 (315.9 ± 73.5 ms). In particular, a greater consistency in self‐paced saccade amplitude was observed (Figure 5) suggesting an improved capacity to actively perform and maintain accurate saccades.

## 3 DISCUSSION

We have described the results of an intense office‐based vision therapy program targeting issues related to visual field defects and co‐existing deficits of binocular vision. Recurring complaints among the patients upon admittance were issues related to ambulation, mainly in environments outside home, and reading problems. The limitation of independence this brings may not only affect quality of life but may also be associated with direct costs related to assistance and transportation. A fairly low percentage of patients spontaneously adapts and develops efficient visual scanning strategies.^7^ It was observed in the current study that patients tended to use compensatory head movements which are markedly slower than eye movements and thus slow the process of continuously updating the global perception of surrounding environment. Excessive head movements may also be disadvantageous in the presence of vestibular issues.

Making eye movements into the nonseeing visual field is a challenge due to the absence of the peripheral preview that normally provides the basis for eye movement programming.^8^ The VTP targeting saccade performance is initially a considerably active and conscious process that aims to reach automaticity as far as possible. Our eye movement analysis showed an improved symmetry in performance for right‐ versus leftward saccades, improved consistency of self‐paced saccades, and less variable fixation behavior suggesting an enhanced automaticity. These skills are critical in the process of maintaining an updated and stable spatial awareness of the surrounding environment .^9^ Our observations were accompanied by improvements in the reading‐related tests, and the patients' reporting enhanced performance and confidence in activities.

A further aspect for efficient eye movements is related to basic visual functions such as visual acuity and binocular coordination. For one of the patients, the spectacle prescription had to be updated for an appropriate visual acuity at far and near. The start of the VTP was postponed until the glasses had been dispensed. Two patients had significant deficits in fusional vergence and vergence flexibility. These functions are needed for the coordination of eye position to maintain clear single vision and for adequate saccade programming. Subsequently, these are important cornerstones for efficient eye movements and compensatory scanning strategies.^10^ Our findings underline the importance of considering optometric examination at an early stage.

## 4 SUMMARY

These case studies show the potential of an intense vision therapy program for restoring objectively measurable visual functions and documented improvements in activity‐based performance and symptoms. The study has limitations in terms of the number of participants and age range, and the outcomes are therefore not generalizable. We still consider the findings to warrant further exploration regarding methods for pre‐ and postevaluation of vision therapy in patients with acquired brain injury.

## CONFLICT OF INTEREST

None declared.

## AUTHOR CONTRIBUTION

IA and AH: performed the clinical examination and vision therapy program, collected and compiled patient information and data, and critically reviewed the manuscript. JJ: performed eye movement recordings and analysis, compiled data, and drafted the manuscript.

## APPENDIX A

**Table A1:** Test paradigms for eye movement testing

Test	Stimuli	Instruction	Measures
Fixation	One red dot, diameter 5 mm, subtending approximately 0.5 degrees. Presented for 15 s at the center of the computer screen	Please look at the red dot as accurately and stable as possible for as long as it is visible	Confidence ellipse area
Pro‐saccades	The starting position was a black cross at the center of the computer screen. Red dots were presented one at a time at a position ±2, ±4, ±6 or ±8 degrees to left or right of the center cross	Please look at black cross. Once you detect a red dot to the left or right please look at it as quickly and accurately as possible. Then return to look at the black cross and wait for new red dot to appear	Latency and gain; mean and standard deviation
Self‐paced saccades	Two red dots presented simultaneously ±6 degrees from the center of the screen for a duration of 30 s	Please look back and forth between the two dots as quickly and accurately as possible for as long as they are visible (30 s)	Inter‐saccadic latency and amplitude of saccades; mean and standard deviation
